# Supplementary material for: Low Levels of Factor H Family Proteins During Meningococcal Disease Indicate Systemic Processes Rather Than Specific Depletion by Neisseria meningitidis
Source: Front Immunol. 2022 May 26;13:876776. doi: 10.3389/fimmu.2022.876776 (PMC9204383; doi:10.3389/fimmu.2022.876776)
Supplement: Supplementary file 1 [file DataSheet_1.docx]

Supplementary Material

# Supplementary figures


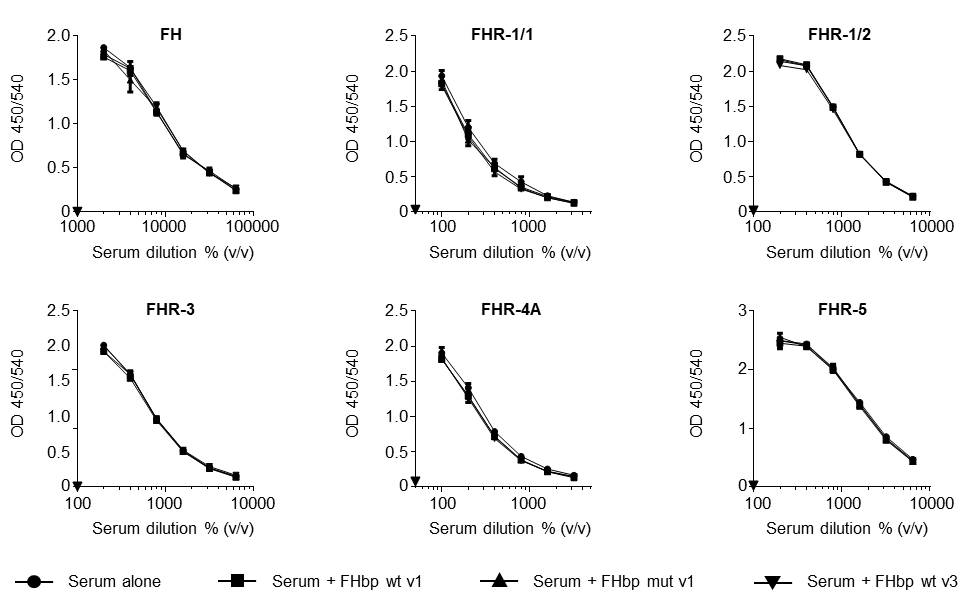


**Supplementary Figure 1 | No fHbp interference in the FH family protein ELISAs**. Normal human serum level measurements of FH, FHR-1/1 homodimers, FHR-1/2 heterodimers, FHR-3, FHR-4A and FHR-5 were tested for interference by recombinant fHbp wildtype of variant 1 (wt v1) and variant 3 (wt v3). Recombinant fHbp that was unable to bind FH was used as control (mut v1). Mean and SD of technical duplicates are shown.

**
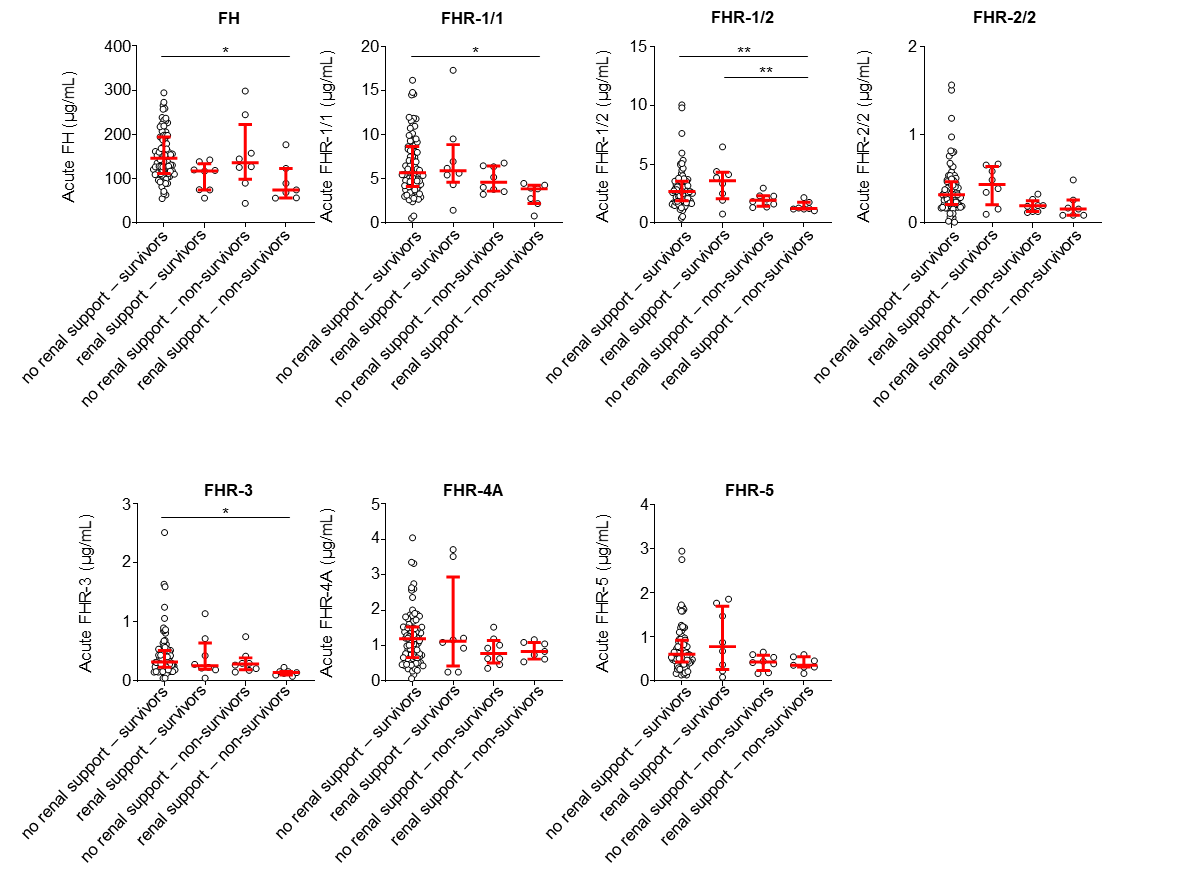
Supplementary Figure 2 | The need for renal support in non-survivors.** Serum levels of FH, FHR-1/1, FHR-1/2, FHR-2/2, FHR-3, FHR-4A and FHR-5 at the acute stage of survivors who did (*n* = 8) or did not (*n* = 83) receive renal support and non-survivors who did (*n* = 7) or did not (n = 8) receive renal support. Statistical significance was tested using a Kruskal-Wallis test, followed by a Dunn’s multiple comparisons test. Lines depict median and IQR. **: *p* < 0.01; *: *p* < 0.05.

**
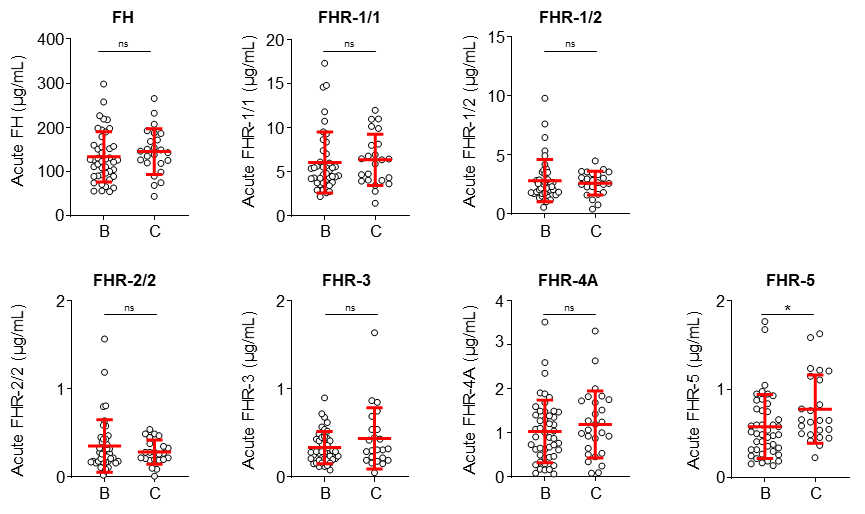
**

**Supplementary Figure 3 | FH family proteins per serogroup.** Serum levels of FH, FHR-1/1, FHR-1/2, FHR-2/2, FHR-3, FHR-4A and FHR-5 according to the meningococcal serogroup, being either serogroup B (*n* = 43) or serogroup C (*n* = 24). Statistical significance was tested using a Mann-Whitney test. Lines depict median and IQR. *: *p* < 0.05; ns = not significant.

# Supplementary Table 1 | Primer/probe sets for *N. meningitidis* quantification.

| Gene | Primer/Probe | Sequence |
| --- | --- | --- |
| *metA* | Fwd | GCGAATTTGCTAATCCTATTTATGTGC |
|  | Rev | AAATTTTGCGCCATTACAGGTG |
|  | Probe | FAM-AACCAGCGCAACGAAAATTGCAA |
| *sodC* | Fwd | GCACACTTAGGTGATTTACCTGCAT |
|  | Rev | CCACCCGTGTGGATCATAATAGA |
|  | Probe | FAM -CATGATGGCACAGCAACAAATCCTGTTT |
| *tauE* | Fwd | TTTCCGATGCTCGGTACAACC |
|  | Rev | CAGGCTTGGTAATGCCACCA |
|  | Probe | FAM-TCATGCCATTGTCTAAGGTTGTTGCCT |
